# Co‐Design for Equity, Inclusion and Engagement: Strategies for Social Prescribing

**DOI:** 10.1111/hex.70779

**Published:** 2026-07-30

**Authors:** Irene Sarasua, Lorelli Nowell, Kimberly Shapkin, Alayne Adams

**Affiliations:** ^1^ Faculty of Nursing University of Calgary Calgary Alberta Canada; ^2^ Ingram School of Nursing McGill University Montreal Quebec Canada; ^3^ Department of Family Medicine McGill University Montreal Quebec Canada

**Keywords:** co‐design, co‐production, equity‐centred design, health equity, integrated care, participatory approaches, social prescribing

## Abstract

**Introduction:**

Social prescribing is increasingly promoted as an integrated care strategy that connects individuals to non‐clinical community resources to improve health and well‐being. Co‐design is frequently recommended for developing social prescribing pathways that are acceptable, feasible and responsive to local needs. However, how co‐design is operationalised in inclusive and equity‐centred ways remains poorly understood. This narrative review synthesises health‐related literature on co‐design strategies that promote equity and foster inclusion in the development of social prescribing initiatives.

**Methods:**

A narrative review of health‐related literature on inclusive and equity‐centred co‐design published within the last 10 years was undertaken. Searches of MEDLINE, CINAHL and Google Scholar (8–28 October 2025), supplemented by reference list screening, yielded approximately 530 records. Sources were reviewed for relevance to the review question, resulting in the inclusion of 52 sources. Findings were analysed using an iterative narrative synthesis approach to identify recurring concepts and patterns, which were integrated into a conceptual framework for inclusive co‐design.

**Results:**

The synthesis identified three interconnected domains of inclusive co‐design: foundational principles, enabling structures and operational practices. Foundational principles establish the values underpinning inclusive co‐design, enabling structures create the conditions for participation, and operational practices translate these commitments into action. Together, these domains formed a conceptual framework for inclusive co‐design in social prescribing.

**Conclusions:**

Inclusive co‐design offers a values‐informed and practical approach for developing social prescribing initiatives that seek to be equitable, relational and responsive to community realities. Although empirical evidence comparing co‐design approaches remains limited, the framework of principles, structures and practices provides guidance for practitioners, researchers, policymakers and community partners. Future research should evaluate co‐design processes and outcomes using measures defined collaboratively with interest holders.

**Patient of Public Contributions:**

No patients or members of the public were directly involved in conducting this narrative review. However, the review was intentionally centred on inclusion, equity, lived experience and participatory approaches. The manuscript draws substantially on literature that foregrounds the perspectives of service users, caregivers, communities and practitioners involved in co‐design processes.

## 1 Introduction

Over the last two decades, increasing attention has been paid to integrated care models that foster collaboration across sectors to address the social determinants of health and advance the Quintuple Aim for Healthcare Improvement: improving population health, enhancing patient experience, reducing costs, supporting provider well‐being and promoting equity [1, 2]. One such model gaining traction in primary care settings in Canada and globally is social prescribing, an approach that connects individuals to non‐clinical community resources to improve health and well‐being [3, 4].

Social prescribing leverages community‐based resources and supports to address people's social needs [3, 4]. Common examples of ‘social prescriptions’ include exercise programs, arts and cultural activities, outdoor recreation, and income or housing assistance [5]. Evidence from numerous studies suggest promising outcomes, including reduced loneliness, improved self‐reported health and quality of life, greater social connectedness, and stronger community ties [6, 7, 8, 9]. Health system benefits may include lower healthcare utilisation, fewer emergency department visits and cost savings [7, 8, 10].

While structures vary, social prescribing programmes are typically organised around a pathway in which a healthcare provider identifies a client's unmet social needs and refers them to a link worker or navigator. This non‐clinical professional supports the client to identify priorities, co‐develop a personalised wellness plan and connect with appropriate community services. Together, the link worker and healthcare provider may monitor progress, address access barriers and track outcomes to ensure the ‘prescription’ remains relevant and effective [11, 12]).

Insights from social prescribing implementation efforts highlight co‑design as a key strategy for developing pathways that are aligned with healthcare and community priorities, available resources and desired outcomes [13, 14, 15]. In healthcare, co‐design refers to a collaborative process in which diverse interest holders, including patients, providers, and community partners, actively participate in designing solutions to health and social care challenges [16, 17]. Although co‐design, co‐creation and co‐production are conceptually distinct, the lack of consensus on their definitions means these terms are often used interchangeably; in this paper, these terms are used broadly to refer to collaborative approaches that engage multiple interest holders in shaping health and social care solutions [16, 17]. (The term *interest holders* is used intentionally, rather than *stakeholders*, to avoid language that some regard as colonial or exclusionary, and to better reflect the relational responsibilities and legitimate interests of those involved in, or affected by, these initiatives.)

Co‐design is recognised for generating innovative ideas, improving the quality and relevance of outputs, enhancing user satisfaction, and promoting equitable engagement and uptake [18, 19]. As such, social prescribing implementation handbooks and other guidance documents consistently recommend co‐design as the preferred method for developing pathways that are acceptable, feasible and responsive to user needs [10, 12, 20].

Despite its prominence in research and quality improvement projects, the application of co‐design in social prescribing remains poorly understood. Historically, few studies have examined co‐design processes in this context, and those that exist are limited in quality [15]. This lack of clarity has led some to describe the implementation of co‐design in social prescribing as a ‘black box’: its relevance is widely acknowledged, yet detailed information about how processes are operationalized is scarce [13, 15]. While a small number of studies have begun to address this gap [13, 14, 21, 22], evidence remains fragmented. In particular, little is known about specific co‐design strategies that foster inclusion and equity‐oriented outcomes in social prescribing and similar integrated health and social initiatives.

Given the broad aims of social prescribing, including advancing health equity, and the recognition of co‐design as a critical component of effective implementation, closer examination of co‐design methods and strategies is warranted.

The purpose of this narrative review is to answer the question: what co‐design strategies promote equity and foster inclusion in the development of social prescribing initiatives? To address this question, we synthesised health‐related literature on inclusive and equity‐centred co‐design, with a focus on processes and strategies transferable to social prescribing contexts. For ease of reading, we use the term ‘inclusive co‐design’ to refer to approaches that aim to achieve these outcomes.

## 2 Methods

A structured narrative review method was undertaken to synthesise health‐related literature on inclusive and equity‐centred co‐design, with the aim of identifying strategies relevant to social prescribing. This method was selected because the review question was interpretive and practice‐oriented rather than focused on estimating the effectiveness of a discrete intervention or exhaustively mapping a bounded field of evidence. The literature on inclusive co‐design spans varied disciplines, settings, methods and source types; therefore, a narrative review provided the flexibility needed to integrate empirical, conceptual and grey literature while retaining an explicit and transparent search and selection process [23].

### 2.1 Search Strategy

Relevant sources were identified through database searches of MEDLINE (OVID), CINAHL (EBSCO) and Google Scholar between 8 and 28 October 2025. Search terms included combinations of *co‐design, co‐creation, co‐production, participatory design, inclusive design, equity‐centerd design, social prescribing, integrated care, health equity* and *patient engagement*. Reference lists of relevant sources were also reviewed to identify additional sources.

### 2.2 Relevance Criteria

Sources were considered relevant to the aim of the review if they: (1) examined co‐design, co‐creation, co‐production or related participatory approaches; (2) focused on co‐design *processes* (rather than solely on outcomes); (3) incorporated an equity lens; (4) were situated within health, social care, community or public service contexts; and (5) were published within the past 10 years. Sources were excluded if they focused solely on technical design methods without attention to participation, inclusion, equity, or relevance to health or social care contexts. Screening was conducted iteratively, consistent with narrative review methodology, with inclusion guided by conceptual relevance to the purpose.

### 2.3 Screening and Selection

A search of three databases yielded approximately 530 records, which were initially screened using title and abstract review. Full texts of potentially relevant sources were then assessed against the relevance criteria. Of the database records screened, 36 sources met the relevance criteria. In addition to database searches, the reference lists of these 36 sources were screened to identify additional relevant sources. Through this iterative process, 16 additional grey literature sources were identified. These sources were made up of policy documents, organisational reports, guidance materials and online toolkits.

In total, 52 sources were included in the final synthesis (36 from database searches and 16 identified through reference screening). The literature comprised a diverse mix of source types, including empirical studies (*n* = 8), review articles (*n* = 8), concept/practice paper (*n* = 5) books/book chapters (*n* = 15) and grey literature (reports, toolkits and other conceptual materials) (*n* = 16). This distribution reflects the heterogeneous and practice‐oriented nature of the co‐design field.

### 2.4 Data Analysis

**Figure 1:**
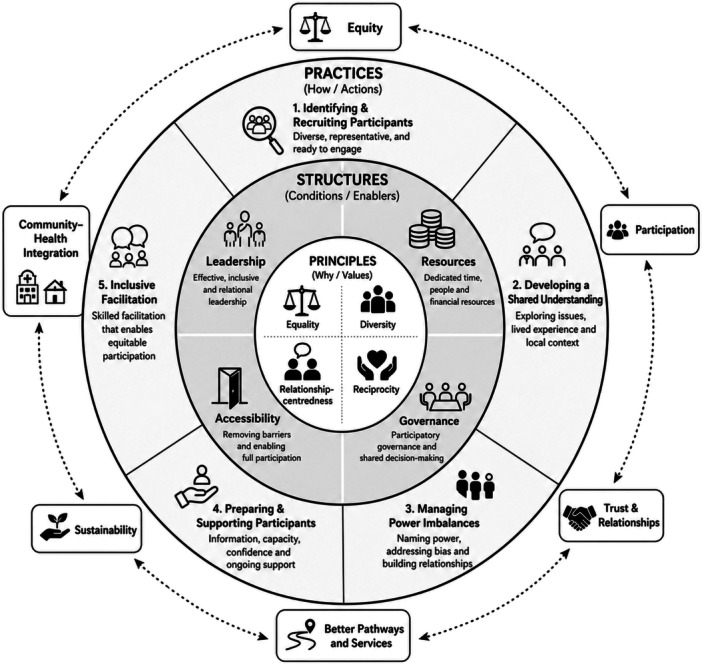
Conceptual framework for inclusive co‐design. The three inner concentric circles represent the domains identified in the review, namely principles, structures and practices. The outer ring reflects the broader aims and outcomes of inclusive co‐design in integrated health and social initiatives.

Analysis was conducted using an iterative narrative synthesis approach. Sources were read closely and compared to identify recurring concepts and patterns related to inclusive co‐design. Rather than formal coding, concepts were grouped through repeated review and synthesis, and organised into a conceptual framework (Figure 1). This process emphasised thematic organisation and conceptual integration across sources, consistent with narrative review approaches [23]. Formal quality appraisal and meta‐analysis were not undertaken. Instead, the review sought to identify patterns, areas of convergence and gaps in the literature to provide practical guidance for those designing social prescribing initiatives.

The authors declare the use of an artificial intelligence‐assisted tool (ChatGPT; OpenAI) to assist in translating narrative findings into a visual schematic illustrating the conceptual map for inclusive co‐design (Figure 1). The tool was used for visual design only and did not generate or influence the content of the manuscript.

## 3 Results

The analysis identified three overarching themes, conceptualised as domains of inclusive co‐design: foundational principles, enabling structures and operational practices. Principles provide the values and ethical commitments that shape strategic intent; structures create the organisational and relational conditions that enable inclusive co‐design; and practices translate these commitments into concrete action. Together, they frame inclusive co‐design as a values‐informed process that relies on supportive conditions and deliberate action rather than a discrete set of methods or activities.

### 3.1 Domain 1: Foundational Principles of Inclusive Co‐Design

Principles are the underlying values and ethical commitments that shape the direction and intent of inclusive co‐design processes. These principles influence how co‐design is conceptualised, structured and enacted. In this review, four foundational principles of inclusive co‐design were identified: *equality*, *diversity*, *relationship‐centeredness* and *reciprocity*.

### 3.2 Equality

Equality in co‐design means that all participants have equal power, voice and value throughout the process. For co‐design to be inclusive, participatory and engaging, those involved must be regarded as capable contributors whose input is genuinely valued [15, 24, 25, 26]. This principle is rooted in social justice and human rights frameworks, viewing co‐design as ‘a moral endeavour that recognises the legitimate knowledge and lived experience of people who typically have services “done to” them’ [27, p. 3]. This perspective challenges paternalistic models by positioning service users as ‘experts by experience’ and essential partners in decision‐making around health innovations [25, 28].

Equality requires the rejection of hierarchical assumptions and the adoption of an asset‐based approach, where no group or individual is considered more important than another and all participants are recognised as bringing valuable assets, such as skills, time and experience, that enrich the process [24, 26]. Importantly, equality extends beyond participation in co‐design activities to the outcomes valued by interest holders. Respecting all contributions includes prioritising outcomes that matter to all participants, not solely those valued by health organisations, such as clinical metrics [26, 29, 30].

### 3.3 Diversity

Diversity in co‐design refers to the intentional inclusion of varied identities, experiences, perspectives and ways of knowing. Co‐design initiatives should proactively bring together and leverage multiple viewpoints to generate ideas that might otherwise remain unexplored [24, 25, 26, 31]. The more varied the input, the greater the likelihood of developing approaches that are relevant, acceptable and feasible for diverse interest holders [32].

While diversity enriches co‐design, it also introduces complexity. Authentic inclusion requires deliberate effort and readiness to navigate tensions, uncertainty and competing priorities [31]. One critical source of complexity is intersectionality: the recognition that overlapping social identities such as race, gender and socioeconomic status shape unique experiences of privilege and marginalisation within systems of power [33]. Applying an intersectional lens in co‐design helps ensure that strategies account for intersecting realities and identities across populations [34].

Diversity should not be limited to service users or clients. It must also encompass healthcare providers and organisational partners involved in co‐design. Assuming that providers are a monolith and thus overlooking their diversity across intersecting identities may undermine efforts to bring varied perspectives to the table. This dimension appears underexplored in the literature reviewed.

### 3.4 Relationship‐Centeredness

At the core of co‐design processes aimed at fostering long‐term engagement are relationships. Individuals are more likely to participate actively when trust and familiarity have been established among those involved. Evidence suggests that interpersonal connections are not only beneficial but essential to effective co‐design, hence their prominence as a recurring theme across the co‐design literature [15, 16, 19, 35, 36]. Co‐design often depends on regular, long‐term relationships between service providers and users, grounded in open dialogue, shared resources and mutual responsibility [16].

Relationship‐building intersects with other principles such as equality and reciprocity. Building a culture of equality begins with people getting to know one another, which opens the door to trust and mutual learning [26, 35]. In this way, relationships function not only as a value, but also as a mechanism for co‐design. Relationships also appear to matter beyond the design phase and are considered important for sustaining innovations over time [37]. Strong, resilient relationships help projects survive challenges such as funding cuts, leadership changes or shifting organisational priorities [37].

### 3.5 Reciprocity

In the context of co‐design, reciprocity refers to ensuring that participants receive something in return for their contributions. It builds on the human desire to feel needed and valued and emphasises creating mutual benefit and shared responsibility among all interest holders [26]. Reciprocity fosters trust, strengthens social bonds, and is considered a critical driver of engagement and motivation in co‐design processes [26, 27, 32, 38].

Reciprocity can take different forms, including ‘material incentives’, such as payment for participation; ‘solidary incentives’, such as social connection and fellowship; and ‘purposive incentives’, such as commitment to a shared mission or cause [37]. Grinspun et al. [39]'s *Social Movement Action Framework* offers an important perspective on purposive incentives through its discussion of ‘intrinsic motivation’, defined as the ‘voluntary engagement of individuals, groups, and/or organisations that share a passion for the need for the change’ (p. 416). While intrinsic motivation is vital, it does not diminish the importance of material incentives, which can enable participation among individuals who might otherwise be excluded. These forms of reciprocity are not mutually exclusive; rather, they can coexist to support sustained engagement.

### 3.6 Domain 2: Enabling Structures of Inclusive Co‐Design

Enabling structures are the formal and informal arrangements that create the conditions for inclusive co‐design. These structures influence how opportunities for engagement are organised, supported and sustained. In this review, four enabling structures of inclusive co‐design were identified: *effective leadership*, *dedicated resources*, *accessibility* and *participatory governance*.

### 3.7 Effective Leadership

Effective leadership in inclusive co‐design involves both *who* participates, and *how* they lead. Meaningful and sustained engagement of formal and informal leaders across diverse interest holder groups can stimulate participation and build confidence that the co‐design process will lead to real change [15]. Without active involvement and visible support from those with decision‐making authority or influence over resources, co‐design efforts may lose momentum, and participants may disengage [18, 31].

Equally important are leadership attitudes and approaches. Leadership that aligns with co‐design principles tends to be distributed, inclusive and relational in nature [15, 40, 41, 42]. Distributed leadership shifts authority from a single individual to a team, fostering collective ownership [43]. Inclusive leadership values diversity, promotes belonging, and creates conditions where all people can contribute to decisions [40]. Relational leadership emphasises trust and collaboration through open questioning, honest dialogue and shared reflection [41]. In social prescribing contexts, leaders who ‘surrender autonomy and embrace adaptability’ fostered a sense of ownership among community members and frontline partners [15, p. 7].

### 3.8 Dedicated Resources

Co‐design requires both human and financial resources. Human resources include time for planning, participation and follow‐up. Financial resources cover participant compensation, facilitation, venue costs, transport, childcare, translation and accessibility needs. Adequate resourcing is closely tied to the principle of reciprocity and is essential for equitable participation [24, 26, 37].

For salaried participants, such as healthcare providers or organisational staff, support may include protected time, flexible scheduling and recognition that co‐design is core work rather than an optional add‐on [18].

For non‐salaried participants, compensation should reflect time, effort, expertise and the value of lived experience [33]. This is particularly important where sharing experiences involves emotional labour or where unpaid participation would create barriers for low‐income participants [33]. Key considerations in determining compensation include the local living wage, barriers faced by low‐income participants, research ethics standards when co‐design is part of a research protocol and local guidelines for similar compensation models [24, 33, 44]. In this way, fair compensation functions as both a practical support and an equity strategy.

### 3.9 Accessibility

Accessibility must be built into the planning and structure of inclusive co‐design activities [24]. Removing barriers helps ensure that all participants have an equal opportunity to engage meaningfully [45]. Accessibility begins with understanding participants’ needs and making appropriate arrangements. This may include physically accessible convenient locations, transport support, childcare, meals, plain‐language materials, translation, interpretation, digital access and culturally relevant communication methods [45]. Timing and duration of activities should also support participation rather than exclude those with caring responsibilities, employment constraints, health needs or transportation limitations [33, 45, 46].

Accessible communication is equally important. Flexible approaches such as visual materials, audio resources, summaries, one‐to‐one conversations and multiple opportunities for feedback can help ensure that information is understandable and usable by all participants [26, 47]. Facilitation practices play a key role in enabling accessible communication by actively encouraging participation, adapting communication styles, and creating space for diverse forms of expression and engagement [26]. Accessibility should therefore be understood not as a single adjustment, but as a process‐wide commitment embedded throughout the design and facilitation of co‐design activities [26].

### 3.10 Participatory Governance

Governance, while closely related to leadership, is distinct in function. Whereas leadership concerns vision, culture and relational dynamics, governance concerns how decisions are made, how accountability is maintained, and who holds authority throughout the co‐design process.

Participatory governance supports diverse representation across the full continuum of co‐design, from planning and recruitment to implementation and evaluation [25]. One practical mechanism is the creation of a representative steering committee, advisory group or monitoring body that includes service users, community organisations, providers and other relevant interest holders [13, 14, 48]. Such structures can help formalise accountability, reduce blind spots associated with homogeneous decision‐making groups, and ensure that principles of equality, diversity and reciprocity are reflected in how the work is governed. Participatory governance may be especially important in social prescribing, where collaboration across sectors requires ongoing negotiation of priorities, responsibilities, and resources.

### 3.11 Domain 3: Operational Practices of Inclusive Co‐Design

Operational practices are the concrete methods and activities through which inclusive co‐design is implemented in practice. These practices translate principles and structures into meaningful opportunities for participation, inclusion and engagement. In this review, five operational practices of inclusive co‐design were identified: *identifying and recruiting participants*, *developing a shared understanding*, *managing power imbalances*, *preparing and supporting participants* and *inclusive facilitation*.

### 3.12 Identifying and Recruiting Participants

Identifying the right mix of participants is a critical first step to ensuring that those involved in, and affected by, an initiative are adequately represented. This process typically involves two stages: defining desired participant characteristics and recruiting individuals who reflect the desired mix. When considering participant characteristics, attention may be given to dimensions such as race, ethnicity, age, gender, sexual orientation, religious beliefs, economic status, physical abilities, geography and life experience, alongside professional roles and community connections [49]. Sibley et al. [34] suggest using intersectionality‐based reflective questions to guide decisions about who should be involved in knowledge translation projects. While it is rarely possible to represent every intersecting identity within a co‐design team, intentional recruitment can broaden the range of perspectives involved. Additional voices may also be incorporated through patient narratives, focus groups, surveys or community consultations.

Useful approaches to identifying participant characteristics include stakeholder mapping, community asset mapping, service utilisation data, census information, patient councils, advisory groups and partnerships with community‐based organisations [13, 25, 50, 51]. A key advantage of stakeholder mapping is its ability to identify both participant groups and specific individuals [13].

Recruitment strategies may include targeted outreach through trusted networks, community champions, newsletters, social media, or local organisations [13, 46]. Participant selection should consider attitudes and readiness for collaborative work. Qualities such as openness, empathy, communication skills, willingness to listen, and respect for diverse perspectives may support constructive engagement [52].

### 3.13 Developing a Shared Understanding

Developing a shared understanding means beginning co‐design with a collective exploration of the issue, grounded in lived experiences and local context, before moving prematurely to solutions [53, 54, 55]. This practice can help prevent superficial problem framing and outputs that fail to address root causes. Developing shared understanding can also strengthen relationships and psychological safety by creating space for participants to listen to one another's experiences and priorities before discussing solutions [56].

A range of tools support this process. These include storytelling, experience mapping, observation, narrative collection, journey mapping and contextual enquiry [31, 53, 57]. Problem‐framing tools such as the five whys, challenge canvases or root‐cause analysis may help groups move from symptoms to underlying issues [58, 59].

### 3.14 Managing Power Imbalances

Managing power imbalances is closely linked to the principle of equality. Differences in status, expertise, confidence, language, institutional authority and social privilege can shape who speaks, who is heard and whose ideas carry weight. Without deliberate attention, co‐design may reproduce rather than challenge existing inequities. Three broad strategies for managing power imbalances emerged: *naming power dynamics*, *confronting biases* and *building relationships*.

#### 3.14.1 Naming Power Dynamics

Naming can help make implicit hierarchies visible. This may include carefully designed exercises to help participants recognise how power and privilege influence the co‐design process and outcomes. Intentional Futures [60] recommends asking critical questions within steering committees to anticipate and plan for uneven power dynamics and introducing tools that encourage participants to ‘cede’ or ‘take’ power during discussions. Other strategies include establishing group norms that acknowledge historic power imbalances and creating space to discuss privilege, exclusion or mistrust in constructive ways [33, 60]. Reflective exercises such as the *intersectionality wheel* and *power self‐reflexive questions* help participants examine areas of advantage or disadvantage and renegotiate assumptions [33, pp. 19–21].

#### 3.14.2 Confronting Biases

Biases, or unfair preferences or prejudices, can significantly undermine co‐design by privileging familiar ideas and limiting diverse perspectives [32]. Confronting biases may help participants reflect on assumptions that shape judgments and interactions. Approaches described in the literature include positionality exercises, perspective‐taking, identity reflection and structured bias training [32, 33, 61, 62, 63]. Kepinski and Nielsen's [64] *The Inclusion Nudges Guidebook* offers a vast array of strategies to change perceptions and reduce unconscious biases and stereotypes.

#### 3.14.3 Building Relationships

Relationship‐building can reduce assumptions and support trust. Activities such as guided introductions, shared meals, storytelling, creative warm‐ups, nature walks or informal conversations may help humanise participants beyond formal roles and titles [65].

### 3.15 Preparing and Supporting Participants

Preparing participants for co‐design and supporting them throughout the process emerged as a recurring theme, particularly in projects involving vulnerable or structurally marginalised groups [24, 26, 66]. Meaningful participation often depends on access to information, confidence and ongoing support [24, 26, 35].

Preparation may include plain‐language information about the project, session goals, schedules, expectations, terminology and opportunities to ask questions in advance. Pre‐session summaries or reflective exercises can help participants feel ready to contribute [46, 67].

Ongoing support may involve regular check‐ins between sessions to clarify processes, answer questions, address concerns and provide one‐on‐one opportunities for those less comfortable speaking in groups [24]. Such supports may be particularly important for participants unfamiliar with professional or institutional environments.

Preparation is not limited to service users. Healthcare providers and organisational staff may also need support to work collaboratively with communities, share decision‐making and value experiential knowledge alongside professional expertise [31, 68, 69].

### 3.16 Inclusive Facilitation

Inclusive facilitation is the intentional process of guiding co‐design activities in ways that support equitable participation, psychological safety and meaningful contribution from all participants [70]. Effective facilitators combine design expertise with strong communication and relational skills, enabling them to tap into the collective creativity of the group [67].

The literature points to two complementary dimensions: what facilitators do, and how they do it. The ‘what’ includes practical engagement strategies such as small‐group work, brainwriting, visual prompts, voting systems, role play, prototyping, written feedback and multiple ways of contributing verbally or non‐verbally [32, 57, 67, 71].

The ‘how’ includes active listening, empathy, neutrality, curiosity, flexibility and the ability to navigate conflict constructively. Inclusive facilitators monitor airtime, encourage quieter voices, validate contributions, address harmful dynamics when needed, and remain attentive to group energy and emotional safety [33, 46, 72, 73].

Skilled facilitation may be one of the most important practical determinants of whether co‐design processes feel tokenistic, extractive, and exclusionary, or meaningful, respectful, and productive.

### 3.17 Conceptual Framework for Inclusive Co‐Design

A visual representation of the conceptual framework for inclusive co‐design is presented in Figure 1. The framework illustrates the interconnected nature of the three domains identified in this review: foundational principles, enabling structures and operational practices. These domains are depicted as three concentric circles, reflecting how values, conditions and actions interact to support inclusive co‐design. The outer ring represents the broader aims and outcomes associated with inclusive co‐design in social prescribing, illustrating that these domains collectively contribute to system‐level goals such as equity, sustainability and community–health integration.

## 4 Discussion

This review set out to identify strategies that promote inclusive and equity‐centred co‐design that can inform social prescribing initiatives. The results suggest that inclusive co‐design is best understood not as a single method, but as a multi‐dimensional process shaped by foundational principles, enabling structures and practical actions. Together, these domains offer a framework for designing participatory processes that are more likely to be ethical, feasible and responsive to community realities.

### 4.1 Evidence Gaps and Why Co‐Design Still Matters

Although co‐design is widely promoted across health and social care, the empirical evidence base remains underdeveloped. Current literature offers limited insight into which approaches are most effective, under what conditions or through which mechanisms co‐design influences outcomes [29, 31, 74, 75]. Much of the available literature consists of case studies, descriptive reports, implementation guides and conceptual discussions rather than rigorous comparative evaluations [31].

The limited state of the evidence, however, does not negate the value of co‐design. Authors argue that co‐design can be justified not only instrumentally, but ethically, because it reflects principles of participation, inclusivity, democracy and shared decision‐making [31, 74]. In this sense, co‐design is not solely a technique for improving services, but also a way of redistributing voice and influence within systems that have historically privileged professional and institutional expertise.

For social prescribing, this distinction is particularly important. Because social prescribing often seeks to address social needs, community connection and inequities, it would be contradictory to develop such initiatives through processes that exclude the very people most affected.

### 4.2 Defining Success in Co‐Design: Whose Values Count?

A critical yet underexplored dimension of co‐design evaluation is the question of who determines what success looks like. Discussions of evaluation often focus on methodological challenges, such as how to measure trust, inclusion, empowerment or participation [29, 31]. Less attention has been paid to the prior question of whose values shape the selection of outcomes.

If the democratic ethos of co‐design is to be taken seriously, then those involved in co‐design should also help define how success is judged. This means moving beyond assumptions that health systems alone determine value. In social prescribing, conventional indicators such as referral numbers, service uptake or healthcare utilisation may be useful, but insufficient. Participants may value different outcomes, including confidence, belonging, dignity, trust, reduced isolation, stronger relationships or a greater sense of agency.

Evaluation frameworks that fail to include such outcomes risk narrowing the meaning of success and reproducing existing power hierarchies in knowledge production.

### 4.3 Risks of Tokenism and Value Co‐Destruction

The language of co‐design is increasingly popular, but practice does not always reflect principle. Where participation is superficial, tightly controlled or primarily symbolic, co‐design may become tokenistic. In such cases, people may be invited into processes without meaningful influence over priorities, decisions or resources.

Tokenistic participation can create harms rather than benefits. These harms may include frustration, loss of trust, emotional fatigue, disengagement, reputational damage and wasted resources. Some scholars describe these unintended negative consequences as forms of ‘value co‐destruction’, in which participation processes undermine rather than create value [31, 35, 75, 76].

### 4.4 Implications for Social Prescribing

Within social prescribing, co‐design is often framed narrowly as a tool for developing referral pathways between healthcare settings and community supports. While pathway design is important, the findings of this review suggest that the potential contribution of inclusive co‐design to social prescribing is considerably broader, extending to how programmes are conceptualised, implemented, evaluated and sustained. First, inclusive co‐design may strengthen implementation by improving alignment between programmes and local realities. By engaging diverse interest holders, including communities, service users and front‐line providers, in defining priorities and shaping interventions, co‐design can help ensure that social prescribing initiatives are responsive to the contexts in which they are delivered. Second, inclusive co‐design may contribute to more meaningful and equitable approaches to evaluation by identifying outcomes that matter to communities as well as organisations. Third, inclusive co‐design may help support the sustainability and scaling of social prescribing initiatives by building ownership, trust and cross‐sector relationships among those involved. These relational dimensions are critical in initiatives that depend on collaboration across health, community and social sectors [77].

Additionally, inclusive co‐design may also support more innovative models of social prescribing, including navigators who work across both healthcare and community settings, peer‐led referral models, or bi‐directional referral systems in which community organisations also connect people to healthcare services when needed.

Importantly, the potential contribution of inclusive co‐design must be considered alongside critiques that frame social prescribing as a largely transactional model, one that risks individualising social need without addressing the broader conditions that produce it [77]. Mulligan and Bloch [77] call for a shift away from ‘prescribing’ social activities toward more relational and co‐creative approaches that emphasise collaboration, community capacity and systemic change. Inclusive co‐design aligns with this orientation by involving diverse interest holders in shaping priorities and solutions, and by fostering relationships that can lay the foundation for broader, concerted action across sectors. In this sense, inclusive co‐design may help move social prescribing beyond isolated interventions toward more coordinated efforts that engage with the social and structural determinants of health.

At the same time, these contributions must be balanced against the risks of tokenism and value co‐destruction discussed above. If poorly implemented, co‐design in social prescribing may reproduce existing power imbalances or place additional burdens on communities without meaningful influence or change. Without adequate resources, leadership commitment and attention to equity, co‐design risks reinforcing rather than challenging inequities. This underscores the importance of approaching co‐design as a values‐informed and structurally supported process, rather than a set of participatory techniques.

### 4.5 Implications Beyond Social Prescribing

Although this review was motivated by social prescribing, the findings are relevant to many integrated care and community health initiatives. Across sectors, organisations increasingly seek to collaborate with communities while addressing inequities, fragmentation and declining trust. The framework of principles, structures and practices may therefore provide useful guidance for wider participatory reform efforts.

### 4.6 Strengths and Limitations

A strength of this review is its synthesis of diverse literature into a practical framework that may assist practitioners, researchers, policymakers and community partners. By drawing from health, community and participatory design fields, the review captures a broader range of insights than a topic‐restricted review might allow.

Several limitations should be acknowledged. As a narrative review, the study did not involve formal quality appraisal or exhaustive systematic searching. Relevant literature may therefore have been missed. In addition, the evidence base itself remains heterogeneous and often descriptive, limiting confidence in causal claims about effectiveness. The framework presented should therefore be interpreted as a synthesis of recurring patterns rather than a definitive model.

### 4.7 Future Research

Future studies should move beyond asking whether co‐design is beneficial in general terms and instead examine which approaches work, for whom, in what contexts and why. Comparative evaluations, realist approaches, developmental evaluation and participatory action research may be particularly valuable. Greater attention is also needed to outcomes defined by communities, long‐term sustainability, resource requirements and the experiences of populations most often excluded from participatory processes.

## 5 Conclusion

This narrative review identified three interconnected domains that appear central to inclusive co‐design: foundational principles, enabling structures and operational practices. Together, these domains suggest that co‐design requires more than the use of participatory methods alone. It depends on values such as equality, diversity, relationship‐centredness and reciprocity; organisational conditions such as leadership, resources, accessibility and participatory governance; and practical approaches that enable diverse people to contribute meaningfully.

For social prescribing initiatives, these findings are especially relevant. If social prescribing aims to address social determinants of health, strengthen community connection and advance equity, then the processes used to design and implement such initiatives should reflect those same aims. Inclusive co‐design offers one pathway for aligning process with purpose.

Although empirical evidence comparing co‐design approaches remains limited, the ethical and practical rationale for continued investment is strong. Future research should evaluate co‐design processes and outcomes more rigorously, with particular attention to context, power, sustainability and outcomes defined collaboratively with interest holders.

Used authentically, co‐design has the potential to move social prescribing beyond referral pathways alone, toward more relational, equitable and transformative partnerships between health systems and communities.

## Author Contributions

**Irene Sarasua:** conceptualisation, writing – original draft, methodology, validation, writing – review and editing, data curation. **Lorelli Nowell:** writing – review and editing, methodology, supervision. **Kimberly Shapkin:** methodology, writing – review and editing, supervision. **Alayne Adams:** methodology, writing – review and editing, supervision.

## Conflicts of Interest

The authors declare no conflicts of interest.

## Data Availability

No new data were generated or analysed in this study. This paper is based on a review and synthesis of existing published literature.
